# Locally led adaptation: Promise, pitfalls, and possibilities

**DOI:** 10.1007/s13280-023-01884-7

**Published:** 2023-06-07

**Authors:** M. Feisal Rahman, Danielle Falzon, Stacy-ann Robinson, Laura Kuhl, Ross Westoby, Jessica Omukuti, E. Lisa F. Schipper, Karen E. McNamara, Bernadette P. Resurrección, David Mfitumukiza, Md. Nadiruzzaman

**Affiliations:** 1 Living Deltas Hub, Department of Geography and Environmental Sciences, Northumbria University, Newcastle-Upon-Tyne, UK; 2 Department of Sociology, Rutgers University, New Brunswick, NJ 08901 USA; 3 Environmental Studies Department, Colby College, Waterville, ME 04901 USA; 4 Perry World House, University of Pennsylvania, Philadelphia, PA 19104 USA; 5 School of Public Policy and Urban Affairs, and International Affairs Program, Northeastern University, Boston, MA 02115 USA; 6 Griffith Institute for Tourism, Griffith University, Brisbane, QLD 4111 Australia; 7 Institute for Science, Innovation and Society (InSIS), University of Oxford, Oxford, UK; 8 Department of Anthropology, University of Oxford, Oxford, UK; 9 Department of Geography, University of Bonn, Meckenheimer Allee 166, 53115 Bonn, Germany; 10 School of Earth and Environmental Sciences, The University of Queensland, Brisbane, QLD 4072 Australia; 11 Department of Global Development Studies, Queen’s University, Kingston, Canada; 12 Department of Geography, Geoinformatics, and Climate Sciences, College of Agricultural and Environmental Sciences, Makerere University, P.O. Box 7062, Kampala, Uganda; 13 Department of Health, Ethics and Society, Faculty of Health, Medicine and Social Sciences, Maastricht University, Universiteitssingel 60, 6229 ER Maastricht, Netherlands

**Keywords:** Adaptation, Community led, Justice, Local, Locally led adaptation (LLA), Power

## Abstract

Locally led adaptation (LLA) has recently gained importance against top-down planning practices that often exclude the lived realities and priorities of local communities and create injustices at the local level. The promise of LLA is that adaptation would be defined, prioritised, designed, monitored, and evaluated by local communities themselves, enabling a shift in power to local stakeholders, resulting in more effective adaptation interventions. Critical reflections on the intersections of power and justice in LLA are, however, lacking. This article offers a nuanced understanding of the power and justice considerations required to make LLA useful for local communities and institutions, and to resolve the tensions between LLA and other development priorities. It also contributes to a further refinement of LLA methodologies and practices to better realise its promises. Ultimately, we argue that the utility of the LLA framing in promoting climate justice and empowering local actors needs to be tested empirically.

## Introduction

“Adapt Now”, the 2019 flagship report of the Global Commission on Adaptation,^1^ underscored that “people and communities on the frontlines of climate change are often the most active and innovative in developing adaptation solutions yet lack access to the resources and power needed to implement solutions” (GCA 2019, p. 62). The report set the stage for increased political momentum around the concept of locally led adaptation (LLA), and in 2020, the International Institute for Environment and Development (IIED) and the World Resources Institute (WRI) along with other international partners under the auspices of the Commission developed eight principles^2^ for LLA. Over 100 organisations, including donor agencies, non-governmental organisations (NGOs), and grassroots organisations, endorsed the principles, committing to make changes and to strengthening existing efforts to better incentivize the adoption of or support LLA (WRI, 2022). Later, Anne-Marie Trevelyan, the United Kingdom’s International Champion on Adaptation and Resilience for the 26th Conference of the Parties (COP) to the United Nations Framework Convention on Climate Change, whilst committing £45 million (~ USD 54 million) to LLA efforts, asserted that LLA “is crucial to building resilience across the poorest and most climate-vulnerable communities” (ADB 2021, online). More recently at the 27th COP in Egypt, the Step Change initiative between Canada and the Netherlands valuing ~ £17 million (USD 20.5 million) was launched to accelerate equitable and inclusive LLA in the Global South (IDRC 2022).

LLA’s heightened political profile has led to a growing articulation of the concept in the academic and policy literatures. Scholars and practitioners have offered various definitions and have used case studies to move beyond the initial articulation of the eight principles developed by the Commission. Westoby et al. (2021a, p. 2), for example, emphasise that LLA is adaptation that is “controlled by local people, grounded in local realities, ensures equity and inclusivity, and is facilitated by local networks and institutions”. The authors go on to suggest that LLA embodies: (1) decision-making led by local people, local knowledge, and local institutions; (2) measuring success by local framings and realities; and (3) considering inequities and marginalisation at the local level. Soanes et al. (2021, p. 10) see LLA as “local people and their communities having individual and collective agency over defining, prioritising, designing, monitoring and evaluating adaptation actions, and working with higher levels to implement and deliver adaptation solutions”. The Adaptation Fund Board Secretariat (2020) also commissioned a study on LLA, which provided an overview of locally led actions and interventions supported by the Fund, and offered concrete examples of what LLA looks like in practice. Together, these efforts help illustrate that LLA is premised on the idea that adaptation should shift from centring non-local actors, including those representing international organisations (i.e. top-down approaches), to adaptation that is driven by emancipatory local participation (i.e. bottom-up approaches) (Falzon 2021; Olazabal et al. 2021; McNamara et al. 2022). Absent from the discourse so far, however, inequalities may still be reproduced through ‘micropolitics’ at the local level (e.g. see Tschakert et al. 2016) and through existing inequalities between actors involved in adaptation projects.

This emergent narrative encourages bottom-up approaches in order to better facilitate and finance local initiatives. The goal is to enable local communities and institutions to “lead” rather than be tokenistic beneficiaries of adaptation efforts. Despite the enthusiasm around the concept of LLA, there is still relatively little experience with LLA on the ground to date (IIED 2021). A recent review of 374 adaptation projects from across the globe found that 138 of those contained some elements of LLA, whilst only 22 projects contained strong characteristics that enable LLA (Tye and Suarez 2021). These elements or characteristics include flexibility, investments in community leadership and local institutional capacities, and the reinforcement of adaptation across scales and programmes. The review also found that local actors are often involved primarily as recipients—they rarely assume leadership roles throughout the life cycle of the programme or project and are rarely actively involved in various decision-making processes, including about how adaptation funding is allocated and distributed (Tye and Suarez 2021). Similar findings were reported by Eriksen et al. (2021) and in the Working Group II’s contribution to the Sixth Assessment Report of the Intergovernmental Panel on Climate Change (IPCC 2022), which concluded that existing adaptation efforts have been insufficient to engage local entities in meaningful and empowering ways, resulting in maladaptation (New et al. 2022). Despite the interest in local engagement and broad consensus on its importance, the uptake and implementation of LLA as a targeted approach to adaptation appear to be quite complex, deserving critical attention to understand and address them.

This article critically analyses the promise, pitfalls, and possibilities of LLA. We centre Nancy Fraser’s three dimensions of injustice, Jürgen Habermas’ theory of the public sphere, and Steven Lukes’ three faces of power to theorise how power inequalities between and amongst actors, and injustice may persist in LLA. We focus on three aspects of LLA where these dynamics play out: (1) defining “local”, (2) controlling resources, and (3) tracking success. We argue that power and justice are amongst the most critical enablers of successful and effective LLA, factors which have been overlooked in various discourses up to now. LLA has the potential to avoid or address the injustices of climate change and those of mainstream adaptation that have characterised adaptation for decades. As LLA is not yet a norm in on-the-ground adaptation action (Tye and Suarez 2021), this article contributes to a further refinement, unpacking, and clarification of LLA methodologies and practices and to highlighting the opportunities that exist or could be created to better realise its promise.

## Analytical framework

Top-down adaptation approaches can create injustices—they have the potential to further marginalise vulnerable communities when interventions are centred on the priorities and perceptions of donors and elite organisational actors, all whilst excluding local actors from project development and decision-making (Holler et al. 2020; Eriksen et al. 2021). In several cases, local communities have been left to deal with the maladaptive consequences of programmes and projects, which were inappropriate for local circumstances and conditions (Schipper 2020), leading scholars to call for more sustainable adaptation (e.g. see Eriksen and Brown 2011). For example, pastoral sedentarization in the peripheral lowlands in Ethiopia as part of the country’s Climate-Resilient Green Economy Strategy has increased marginalisation, aggravated food insecurity, and worsened vulnerability of pastoralists (Eriksen et al. 2021). Conversely, the channelling of adaptation funds and other resources from wealthy countries to vulnerable countries that contributed little to cause climate change is a mechanism for climate justice (Ciplet et al. 2022).

Fraser’s (2006, p. 171) definition of justice as “parity of participation” is useful for understanding the promise, pitfalls, and possibilities of LLA because it aligns neatly with the language of participatory planning commonly used in adaptation practice but also used as a basis and means of achieving LLA. Fraser (2009) outlines three dimensions of injustice. First, distributive (economic) injustice is produced through class hierarchies where there is unfairness in outcomes, such as payments. Second, recognitional (cultural) injustice is produced through status inequalities where there is inadequate acknowledgement of context, narrative, vulnerability, relationships, or power. Third, representational (political) injustice is produced through misrepresentation or underrepresentation or through mis-framing (exclusion through boundary setting). Considering these three dimensions, one can imagine how LLA could reproduce injustices due to the economic, cultural, and political inequalities between and amongst donors, elite actors in planning and implementing organisations, government officials, local elites, and project beneficiaries.

The possibilities of LLA are, therefore, limited by an assumption that the inequalities between and amongst diverse actors can be put aside for the sake of a more just and equitable adaptation process and outcome. This assumption is reminiscent of an oversight in Habermasian theories of the public sphere. Habermas (1999 [1962]) imagined the public sphere as an arena of discursive debate in which all are welcome to participate and ideas are exchanged mutually amongst parties. Whatever idea triumphs is due to its strength over other ideas presented. However, the discursive possibilities of the public sphere only exist if participants enter the debate on equal footing. Further, it is impossible to bracket the inequalities arising from economic, cultural, and political circumstances (Fraser 2006, 2009) and, therefore, the public sphere always risks reproducing and exacerbating them as more powerful actors dominate the discourse. Such power inequalities have been acknowledged in climate change policy for decades (e.g. Agarwal and Narain 1991).

Given that inequalities are inherent in interactions between and amongst elite organisational actors and marginalised local people and therefore continuously shape the adaptation solutions that are generated, it is important to consider how power is asserted. For this, we turn to Lukes’ (1974) “three faces of power”. The first face is the most public, whereby power is asserted through direct decision-making. For adaptation, this means that those with power and resources explicitly decide what is needed for adaptation and how and where adaptation should be implemented (Eriksen et al. 2021; Falzon 2021). The second face is power that is asserted indirectly through non-decision-making, often shorthanded as agenda setting. For example, adaptation funders’ perspectives on what counts as adaptation or what counts as development might put some interventions “on the table”, whilst others are excluded. According to its ideals, LLA would challenge this face of power, particularly by removing hierarchical planning structures that prioritise external actors’ organisational priorities ahead of what local actors may envisage. The third face is hegemonic or ideological power. Through hegemony, powerful actors influence the wishes and thoughts of others, even causing them to support actions that are not in their best interest. Here, a non-dominant actor might not imagine better alternatives because a dominant actor has foreclosed alternative possibilities. In adaptation, this may involve local actors neglecting to consider interventions that are outside of the scope of what more powerful organisational actors have led them to believe is possible and therefore not voicing their priorities.

**Fig. 1:**
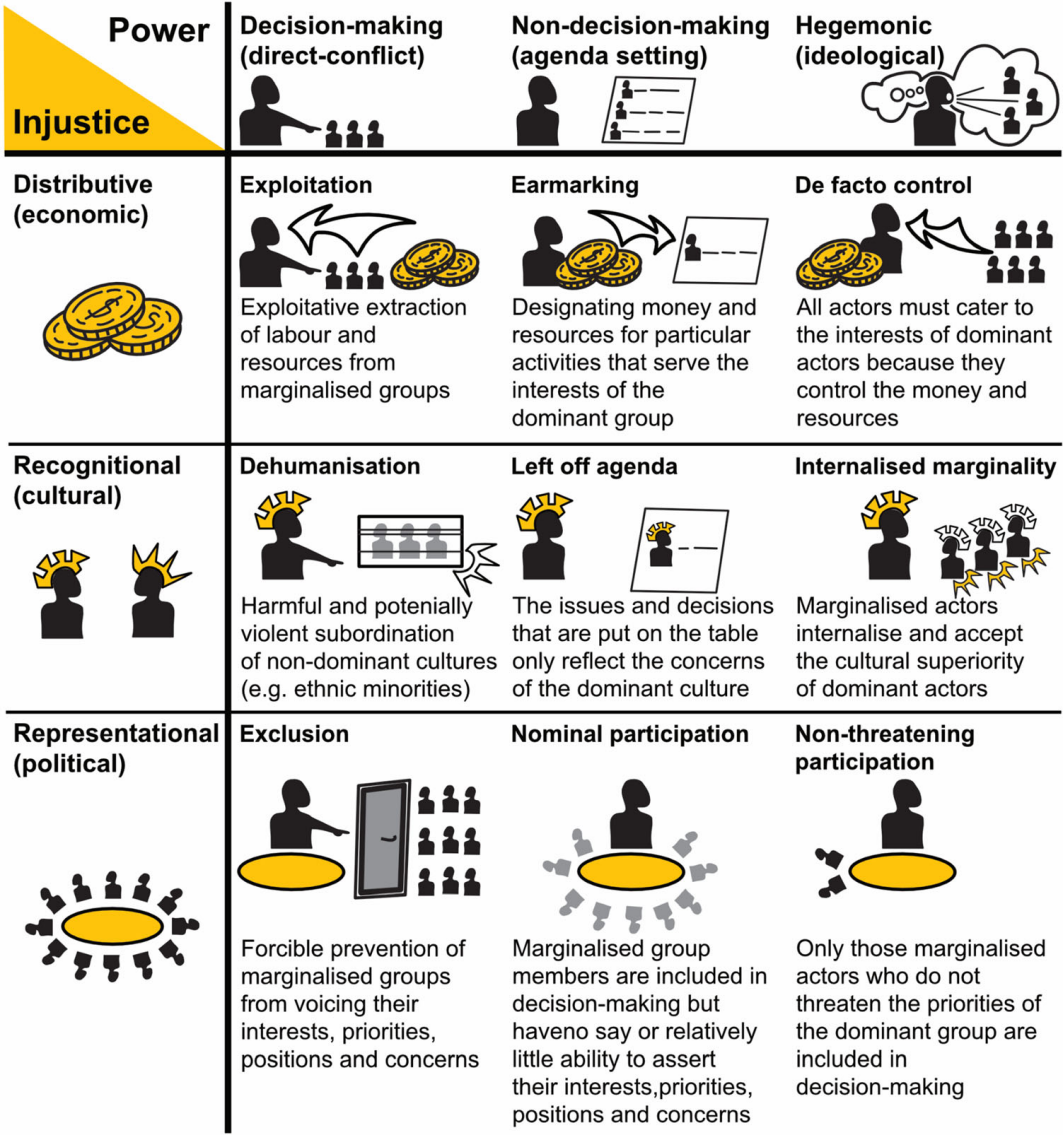
Intersections of Fraser’s dimensions of injustice and Lukes’ faces of power (Source: Authors)

We intersect Fraser’s three dimensions of injustice with Lukes’ three faces of power to identify the ways in which injustice might persist in LLA (see Fig. 1). Whilst direct conflict in adaptation work may take place (Sovacool 2018), agenda setting and hegemonic power are more likely to be enacted across the three dimensions of injustice. Distributive injustice is likely to arise when non-local actors and organisations control the adaptation planning agenda. These non-local actors are those who would typically initiate interactions with local actors and determine how and the circumstances under which interactions will take place. Even if local actors are consulted on agenda setting, the control over resources by non-local actors still gives them de facto control over the discussions and contributes to distributive injustice. Though the ideals of the LLA approach make it unlikely that local actors will be entirely excluded, there may also be a persistent accepted marginality in the interaction whereby inequality in the valuation of cultural traits makes local actors subordinate. Finally, representational injustice risks being reproduced in LLA as non-local actors exert power through agenda setting and hegemonic control. Through agenda setting, local actors may be able to only nominally contribute to the adaptation planning and implementation process because of inequalities already identified in economic and cultural hierarchies. Through the hegemonic control by non-local actors, local actors may only contribute to discussions in a way that is non-threatening to the interests of more powerful actors.

To further clarify our analytical framework, we turn to a working paper from WRI that discusses putting LLA into practice (Coger et al. 2022). Drawing on the examples of mechanisms and approaches for LLA that they provide, we present a hypothetical adaptation project that strives to be locally led and analyse it according to our framework. For our hypothetical project, a development agency in a Global North country has earmarked their funding to a Global South country for water-related climate adaptation work. Additionally, the development agency wants to fund projects that are locally led, so any applicant must work with local people to plan the project. An international NGO (INGO) that works on water issues applies for this funding. First, the INGO engages their country office who then engage with a rural community in which they have worked previously to generate insights on issues around water access. The INGO talks to male community leaders with whom they already have relationships; some of their wives and farmers join a meeting on a day when the INGO’s team can travel to the rural area. At the meeting, the team guides the conversation and leads activities and collaborative mapping exercises, which identify two solutions—the first for reducing the high salinity content of water in tubewells and the second for collecting rainwater for household use. The INGO takes the inputs from the local people back to their office where they realise that they are not sure how they would address the first solution and that they have more experience addressing the second solution that local actors identified. They justify their choice to distribute rainwater collection tanks on the basis that local people were not aware of the costs of reinstalling tubewells or installing a desalination station, whilst the tanks would be much cheaper or cost-effective and more easily implementable within a short project timeline. They write a proposal to apply for funding from the development agency to install rainwater collection tanks in the rural area, receive funding, and install the tanks in places that the local community members select.

In this example, several dimensions of the intersection between power and injustice are illustrated. First, funds were earmarked for a particular issue—water access—by the donors. Dependent on donors for money, the INGO and local actors both had to cater to the donors’ interest in this issue, an instance of distributive injustice as those seeking adaptation for other issues did not have access to those funds. On recognitional injustice, the agenda of the meeting arranged between the INGO and local people was structured to put the concerns of local people on the agenda, but, again, those concerns had to be water related. It is also likely that local people, particularly the women, youth, or the elderly in the meeting, did not feel that they had the agency to articulate all of their concerns and that some concerns were reinterpreted by the INGO. Next, the INGO chose the time of the meeting and controlled who was present. Their prior relationships with local actors, interest in gender equality, and the perception that farmers would be amongst those most interested in water issues all drove their selection of participants. It is possible that these particular local actors may have close relationships with the INGO because they are non-threatening to the organisation’s priorities. Because local people are not a homogeneous, without a process for diverse and wider participation, local ‘micropolitics’ will determine which voices are privileged as representing the local. Finally, the INGO selected between the participants’ solutions based on their own capacity and experience. The INGO, therefore, made the final choice about how the adaptation would take place, making local peoples’ participation at the meeting more nominal than if their priorities had led the decision-making process. What our framework reveals in this hypothetical (but realistic) case is the ways in which power and injustice can persist, even when efforts are made to create locally led projects. This theory-based understanding of the intersections of injustice and power that we present has not yet been widely discussed amongst the proponents of LLA, which we begin to unpack in the next section.

## Evaluating power and injustice in LLA

To better understand how diverse forms of power might produce and reproduce inequality and injustice, we evaluate three critical components of LLA where these dynamics play out: (1) defining “local”, (2) controlling resources, and (3) tracking success. Using the analytical framework we described above, we elaborate on the promise, pitfalls, and possibilities of LLA as a transformative approach to climate change adaptation.

### Defining “local”

*The promise:* A core component of LLA that differentiates it from earlier approaches to adaptation such as “community-based adaptation” is the idea that “local” approaches offer benefits that non-local approaches do not (Vincent 2023). For adaptation to be truly “locally led”; however, we must interrogate the meaning of “local”. In the local food movement in the USA, for example, food that is “local” is that which comes from small farmers and therefore assumed to be tastier, healthier, and more environmentally friendly (Brain 2012, online). In the field of international development, as another example, locally led development, a concept that predates LLA, “refers to initiatives owned and led by people in their own context” (Timson 2021, online). This is important because “when producers and workers take power into their own hands, […] change happens” (Timson 2021, online). In climate adaptation, local engagement makes projects more effective in the long term, more culturally and ecologically appropriate, and more empowering for individuals that are impacted by them (Chu et al. 2019; Ling et al. 2022; Robinson and Butchart 2022). A local perspective also enables the understanding of what people actually do to live with climate change and its emergent discourses instead of focusing merely on what climate change “does” to them (see Castro and Sen 2022).

Often “local” is used in juxtaposition with “global” or “national” to indicate a subnational scale, although, in climate policy, local can even be used to refer to the national context, as climate decision- and policy-making often occur within international institutions. In each of these instances, “local” gives the impression of a smaller, more connected group of people or communities that are closer to the problem of climate change. This emphasis may encourage the exploration of more meaningful, viable, radical, and creative entry points that overcome the inherent issues, gaps, and assumptions around the notion of “community” in adaptation (Yates 2014; Nalau et al. 2015; Ford et al. 2016; Titz et al. 2018; Westoby et al. 2021a).

*The pitfalls*: As LLA has gained popularity, different actors have been using the term quite differently, suggesting a lack of common understanding on the meaning of “local”. At the virtual Gobeshona^3^ Global Conference on Adaptation in 2021, which focused on research into action on LLA, representatives of global climate funds generally related “local” ownership of adaptation projects to autonomy by national governments and institutions over the spending of international adaptation finance. For example, international climate funds depict themselves as promoting local ownership, despite evidence that direct access projects may not always reflect local priorities (Omukuti 2020a,b; Kuhl and Shinn 2022). Representatives from INGOs at the conference presented on LLA projects on which they worked to give community members the greatest possible agency over how the project would be carried out. These projects, however, often sought out “local” inputs at stakeholder meetings as new projects were being planned or where those in attendance were prominent academics and practitioners working on climate and development issues in the country. In each of these cases, “local” was used to refer to people who are quite different from each other and who have varying levels of power, which can create at least two potential challenges in practice, which we will discuss below.

The first challenge is elite capture (e.g. see Nightingale 2017; Omukuti 2020a; Taylor and Bhasme 2021) wherein the power structures at local levels mirror the same power structures of the “haves and have nots” in the wider global capitalist system (Nadiruzzaman and Wrathall 2015; Tschakert et al. 2016). Whilst ideally systemic inequalities will become a driving agenda for local implementers who know first-hand the power dynamics and relations that are embedded in each local context, elite capture persists when there is no strong agenda to tackle it (Tschakert et al. 2016). LLA projects may end up being led by the same few people in the “know” who gain access to adaptation funding in communities (e.g. see Adaptation Fund 2020). The underlying power structures at the local level can be reinforced by community-based organisations or other local mechanisms (Westoby et al. 2020a, 2021a). This also raises three questions: (1) Who has claims to locality? (2) To what extent do elites have the authority to speak on behalf of local communities and institutions? (3) If not the elites, what are the mechanisms for engaging other local people when confronted with the persistent faces of power? Given the varying definitions of “local” already being employed in discussions on LLA, local actors could include anyone from a government representative to the director of an NGO, to a community leader, and to any individual in the project area. In LLA, someone must be identified as “local” who can theoretically help lead the adaptation effort, suggesting that LLA requires specificity on what qualifies an actor as being “local”. Otherwise, the approach is the same adaptation as usual.

The second problem is whether the concept of “locally led” can be reduced to a specific geographical location or set of spatial characteristics (Timson 2020). Scholars studying the local food movement in the USA, for example, have pointed out that people have varying perceptions of the distance that should be considered “local” and that locality can and should not be understood as being equivalent to terms like “organic” that ascribe a value to the quality of the food and/or its production (Dunne et al. 2011; Hand and Martinez 2010). Similarly, in the case of LLA, “local” actors targeted for adaptation interventions have varying spatial characteristics. For example, “community” has often been used by adaptation actors with little critique, but “communities” do not necessarily lend themselves neatly as “clear entities” for intervention (Buggy and McNamara 2016; Titz et al. 2018, Clissold and McNamara 2019). What is needed are more nuanced and dynamic understandings that recognise the various layers and complexity in “local” and “community” framings. The emphasis on “local” may help with this. For example, “whole-of-island” or local ecosystem approaches, embedding adaptation in local institutions such as vocational colleges or working with specific population groups such as women or religious minorities, have all been documented to be entry points that can effectively serve the interests of local groups (Clissold and McNamara 2020; Westoby et al. 2020a, 2021b). When adaptation entry points are not predestined by existing normative constructions of “community” or predefined spatial characteristics, resources, and decision-making power can be invested at different “local” scales.

**Fig. 2:**
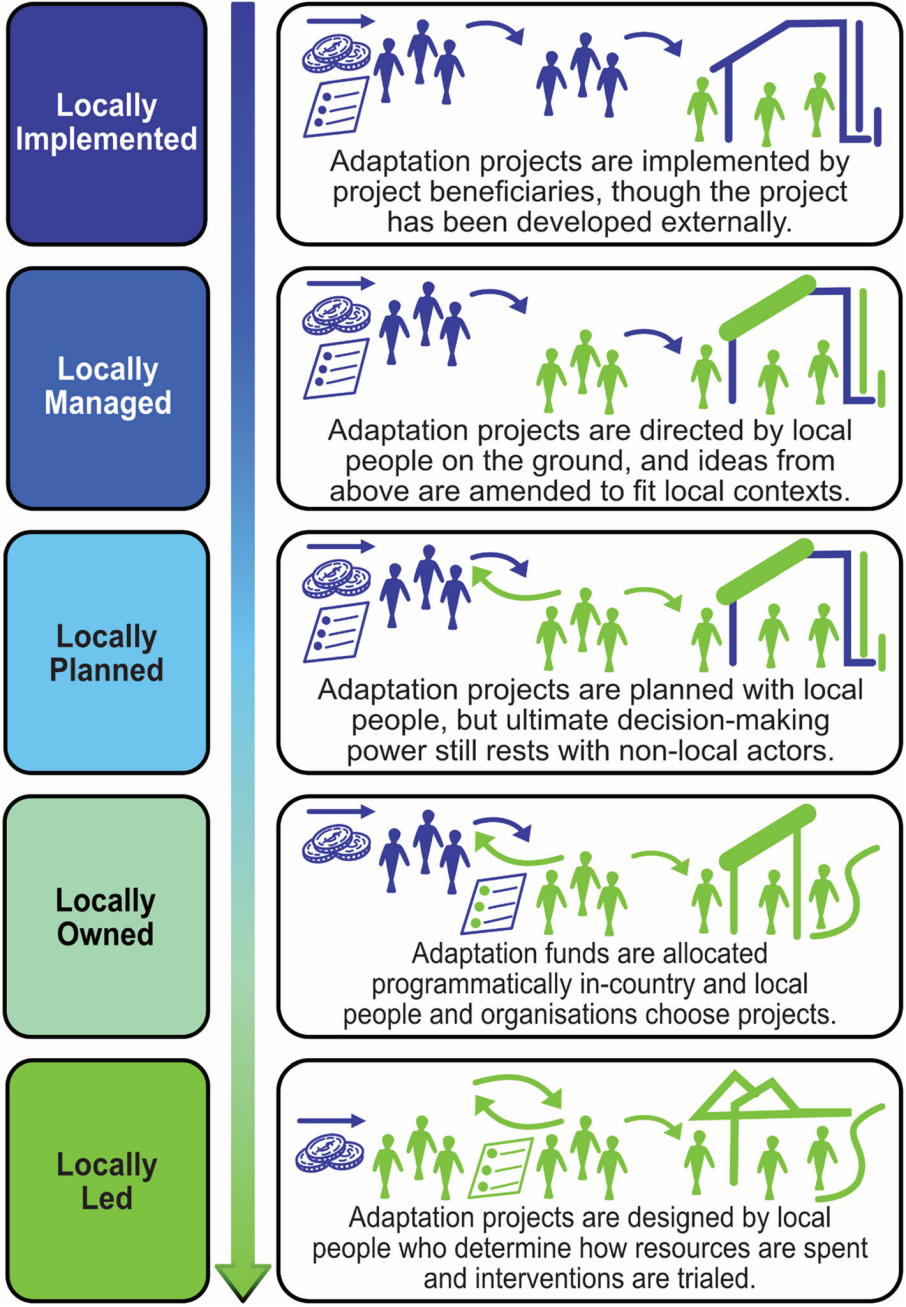
Continuum of local contributions to adaptation programmes and projects (Adapted from USAID 2021)

*The possibilities*: Keeping the intersecting dimensions of injustice and power in mind, we believe that each LLA intervention should specify what “local” and “locally led” means in its context, defined according to scale and desired outcomes. This nomenclature is critical. Improved understanding of the “who” and “what” of adaptation needs critical reflection to ensure that actions are actually locally led. We acknowledge that, as LLA becomes a more prevalent discourse in adaptation practice, there is a risk that its meaning will be diluted to apply to all forms of local engagement in adaptation projects. This risk comes from adaptation actors’ desires to “jump on” LLA as the latest trend to secure funding. There is a danger that existing adaptation projects will be reframed as LLA or be rebranded without necessarily fully embracing an LLA approach (see similar discussions in Robinson 2019). The rebranding issue is already significant in adaptation projects, where this process can lead to increased, rather than decreased, vulnerability (Eriksen et al. 2021). It is, therefore, useful to understand that local engagement in adaptation takes place in a variety of ways along a continuum. Figure 2 illustrates the range of possibilities, beginning with the projects offering the least local agency (top) to those with the most local agency (bottom). The dashed line separates business-as-usual approaches to local inclusion in adaptation (above the line) and more transformative approaches that contribute to local leadership in adaptation (below the line). The arrow is intentionally unidirectional to illustrate a progression that moves towards LLA, leaving behind more top-down approaches. This figure draws from a similar continuum used by the United States Agency for International Development (USAID 2021) that categorises projects as more or less locally led. As the figure suggests local participation in adaptation below the locally led threshold may be steps in building towards LLA and climate justice.

### Controlling resources

*The promise*: LLA comes with the promise that local people can control finance and other resources available for adaptation, implying that they have both easy access to these resources and decision-making power over how they are used. This contrasts the current state of adaptation finance where money and resources are concentrated amongst funders and international organisations, whilst countries, local organisations, and local people have little direct access to funds (Ciplet et al. 2022). To date, the push for LLA has predominantly focused on lack of finance reaching the local level. An IIED research paper reported that only 10% of the allocated climate funds reach the local level (Soanes et al 2017), which has been confirmed by other studies (e.g. Manuamorn et al. 2020; Browne and Razafiarimanana 2022). This trickle-down nature of adaptation finance means that actors at the lower levels of the finance hierarchy experience distributive injustice in funding (Ciplet et al. 2022). As our analytical framework above indicates, when a subset of actors dominate resources, funds can be earmarked for projects preferred by these subset of actors and the monopolisation of funds means that only projects that attract and serve a narrow set of interests are likely to be funded. Even ostensibly objective funding criteria, such as additionality or a clear climate rationale, can mask the hidden politics of adaptation finance decision-making and encourage applicants to “design for the fund” (Robinson 2018; Kuhl 2021; Kuhl and Shinn 2022; Thomas 2023). This is the status quo that LLA seeks to change.

LLA promises to enhance distributive justice by reducing funders’ and international organisations’ control over resources and instead increasing local actor control of them. The LLA programme of the Global Centre on Adaptation (GCA), which “aims to empower local governments and communities through the provision of additional resources and capacity” (GCA 2022, online), calls for significant increases in the volume of devolved and decentralised funding available to local actors to enhance access to resources and power needed to implement adaptation solutions. One of the participants of an IIED podcast on accountability for LLA suggested “finance *is really at the heart of what it takes to get locally led adaptation moving quickly and at the scale that is needed to meet our ambitions over the next 10 to 20 years”* (IIED 2021, online). LLA, therefore, promises to shift a significant degree of power over decision-making from the international and national levels to subnational and other local actors, enabling individuals and communities to leverage their creativity to develop adaptation solutions based on local realities. It marks a growing recognition that “top-down” and externally led approaches are often unsustainable, unjust and even harmful (Eriksen et al. 2021; Forsyth and McDermott 2022).

*The pitfalls*: The greatest challenge to enhancing distributive justice through a locally led approach is overcoming the existing institutional infrastructures and norms that guide the flow of adaptation finance. Trust between those actors that control financial and non-financial resources, and national and subnational actors are often absent. Funders and international organisations are wary of the potential for corruption in administering resources directly to local actors and have mechanisms in place that ensure their control over what they believe to be their “investment” (Falzon 2022). For example, as part of climate funding proposals, applicants must rigorously demonstrate that their organisation’s financial and institutional capacities meet the funding organisation’s standards and that they can responsibly manage the funds. Whilst justified by funders as a means of ensuring quality in project design and due diligence, these criteria along with elements, such as the requirement to demonstrate a strong “climate rationale” and impact pathways, practically prevent local actors from accessing funds directly (Kuhl 2021; Kuhl and Shinn 2022). This raises the question of whether this investment logic, which is based on outcomes, outputs and deliverables typically on 3–5-year timelines, is compatible with successful and effective LLA processes.

Whilst LLA envisions a system of funding distribution that prioritises local access, the ease of access to these funds by local actors is relative and there may be different barriers for different groups of people that must be considered (Holler et al. 2020). This may require developing local capabilities in a way that enhances local actors’ abilities to directly access and control funds based on their own priorities and needs rather than producing the institutional infrastructures that funders expect (Robinson and Dornan 2017; Holler et al. 2020). Furthermore, if LLA were to be carried out based on a model in which national governments receive funding from international sources and then distribute it programmatically, the onus would still rest on national or subnational governments to acquire this funding. This may still result in funding allocations that fail to reach grassroots causes and organisations, with decisions still being taken by external actors and limited engagement of non-government and diverse local actors in project design (Omukuti 2020b).

Whilst LLA purports to serve as a transformational approach to adaptation, funders’ ideas about what transformative adaptation should look like may also create institutional barriers to LLA. In the Green Climate Fund, for example, key indicators for the paradigm-shift potential of a project are its scalability and replicability, placing the transformational potential *outside* the scope of the intervention itself and focusing on how it can be scaled up beyond the local site (Kasdan et al. 2021; Kuhl and Shinn 2022). More broadly, international adaptation finance is often justified due to the demonstration potential of projects, again placing the true value in the scaling up of the intervention (Kalaidjian and Robinson 2022). It is not necessarily clear, however, that LLA is scalable. The very features that define LLA—local control and support, context specificity, and equity and inclusivity (Westoby et al. 2021a)—may be antithetical to scalability. Furthermore, the process of scaling up may undermine all of the benefits of LLA.

*The possibilities*: There are significant improvements that can be made to the existing system for the allocation of international adaptation funding that would better facilitate LLA. This requires that funders abandon their current view of international climate finance for adaptation as an investment and instead, seeing it as resources owed to vulnerable communities according to the principles of climate justice (see Khan et al. 2020). Funding agencies and their intermediaries should develop guidelines and policies to ensure that local actors are not disproportionately burdened with reporting responsibilities that come with fund management.

Existing systems place the onus of alignment with donors’ and external actors’ values and requirements on local actors. Localisation without systematic shifts will merely be a new avenue for donors and external actors to impose the same control over financial resources, which could lead to the exploitative extraction of labour and resources from marginalised groups. Maha Shuayb, Lebanese humanitarian scholar, put it exquisitely in an article about localisation (Shuayb 2022, online):

*“If localisation is to be anything more than just a buzzword and tokenism, it must include people from the Global South, from the conception of an idea right through to execution – whether that’s research, programme response, or policy development. It requires breaking the moulds in the current structures that limit “local” actors like me to the margins: grant eligibility and funding criteria; the application processes; and the institutional and academic hierarchies. These structures remain biased and colonial, and they limit the will, vision, voices, and participation of those who are disadvantaged by the system.”*

According to Sheela Patel, the founder of the Slum/Shack Dwellers International and a critical proponent of LLA, one way of transforming existing power structures “is to have more and more “grassroot leaders” sitting with people who make decisions about money” (IIED 2021, online). NGOs, donors, research organisations and consulting companies must learn collectively about how they might transform themselves so that they are better able to engage with, and be directed by, local expertise as opposed to international consultants and researchers.

We can also imagine more transformative possibilities for resource distribution and control in LLA. For example, consistency and transparency in funding are critical. This would mean ensuring regular flows of money and resources to countries and communities who can count on these resources being available over time and plan accordingly (Robinson et al. 2021; Ciplet et al. 2022). Furthermore, if done properly, restructuring the current international adaptation finance system to one that is oriented towards LLA can also reduce costs by removing the middle players that add overhead into spending (see Robinson and Dornan 2017). As such, a greater percentage of adaptation finance will go directly to local communities and to the adaptation interventions themselves. In all of this, the goal of reducing vulnerability must remain central, as funding flows do not guarantee impact. As such, the assessment of projects must also be reimagined.

### Tracking success

*The promise*: LLA acknowledges that there should be downward accountability where funders and other non-local actors report back to local actors. LLA initiatives could use metrics, evidence, learnings and other outputs of the monitoring and evaluation (M&E) process that resonate with local priorities and understandings and empower local actors to hold project leaders and funders accountable. By offering programmatic funding as opposed to project-based funding, LLA promises greater flexibility in adaptation funding to enable adaptive programme management that prioritises and adjusts to learnings acquired from both successes and failures (Soanes et al. 2021). It also offers an opportunity to empower local people to create success metrics that matter to them. Under LLA, M&E frameworks would be aligned with local priorities including focusing on learning to improve adaptation processes at the local level. Rather than be the passive recipients of programmes designed by others, under LLA, efforts to track successful adaptation could start with questions such as What matters to local well-being, and what are individual and community aspirations? What are the most important measures that capture local aspirations, impacts, social frameworks and economic opportunities? Creative quantitative and qualitative methods, including storytelling, participatory video and photovoice, and collaborative community “atlases of community change” could be used to enable their meaningful contribution to the M&E process and capture local perspectives and lived experiences of LLA interventions (Dilling et al. 2019). National governments would also engage with subnational and local perspectives, including non-governmental actors to select what to report in adaptation communications to the Framework Convention on Climate Change, for example.

*The pitfalls*: Unlike mitigation, which focuses on reducing greenhouse gas emissions, universally applicable “success” measures are not available for adaptation (Dilling et al. 2019). This is primarily because of both the varying baseline conditions and community perceptions and expectations at the local level where adaptation projects are implemented and the fact that adaptation processes are shaped by evolving climatic (and non-climatic) conditions that shift with time and availability of new knowledge and technology (Dilling et al. 2019; Eriksen et al. 2021). Adaptation tracking typically does not engage with what really constitutes “success” and does not address questions, such as “who has a voice in talking about adaptation success” (Dilling et al. 2019, p. 573). Available adaptation approaches executed through planning, implementation and M&E may, therefore, privilege certain worldviews and processes over others, thereby enabling them to define such success (Mikulewicz 2020). As our analytical framework suggests, existing approaches do not give local lived experiences and traditional knowledge the same priority as top-down and expert-driven knowledge. As a result, they create leeway for distributive (ideological and agenda setting power) and representational injustice (exclusion and nominal participation). Currently, the architectures of existing climate adaptation funds put more emphasis on financial and economic outputs and outcomes as opposed to human rights and pro-poor international climate finance, and thereby implicitly favour views in line with economic growth, efficiency, bankability, and private sector commercialisation (Eriksen et al. 2021).

Participatory processes have been extrapolated or co-opted to make them functional for M&E that work for those who provide finance (Nadiruzzaman and Wrathall 2015; IIED 2021). The extensive use of indicators exacerbates this dynamic and tends to be an exercise in reductionism in which indicators are selected that can be measured cleanly and neatly. Project-based M&E approaches include reporting templates that focus on project management activities, outputs such as goods and services delivered, number of beneficiaries, and value for money (Atteridge and Remling 2018). In doing so, M&E approaches often ignore longer-term unwanted outcomes, such as subsequent socio-political relations, resilience, or well-being of beneficiary populations or the impacts on non-beneficiary populations (Eriksen et al. 2021). M&E systems that enable external actors to govern climate actions, whilst placing responsibility for implementing actions on local actors may disempower and marginalise actors and worsen their vulnerability (Scoville-Simonds et al. 2020; Eriksen et al. 2021).

As M&E involves power and decision-making, there are growing calls for changing power structures, accountability systems and transforming the status quo, as there are a dearth of initiatives for creating architectures that will actually do so (IIED 2021). Engaging local actors in evaluation and accountability mechanisms does not guarantee that their voices will not be co-opted. LLA, therefore, faces two critical risks. The first is the probability of being co-opted by existing development agendas, entrenching ideologically driven development models whose core assumptions might be fundamentally at odds with vulnerability reduction and support for marginalised groups (Eriksen et al. 2021). The second risk is the probability of donors and intergovernmental agencies still expecting short-term deliverables and outcomes that often promote success with little regard to tying funding to long-term impact outcomes. Local organisations, as a result, might be deluged with the reporting requirements that overburden their staff and resources (see Robinson and Dornan 2017). These risks need to be minimised or eliminated in order for LLA to address existing distributive injustices in adaptation finance and project implementation.

*The possibilities*: As LLA’s political profile continues to increase and if it is to promote climate justice, donors and other non-local actors will need to collaborate to set up M&E systems that are flexible and open to embracing a plurality of definitions and measures of adaptation success. Such systems arguably should focus on whether diverse local actors have enhanced capabilities in influencing adaptation decisions and enhanced capacities to address climate impacts (Dilling et al. 2019; Holland 2017). M&E should evaluate these markers both for adaptation outcomes and for the project planning and implementation processes that produced them (Groce et al. 2019). Likewise, locally appropriate measures of reporting adaptation “failure” could be helpful, alongside an exploration of the discursive power dynamics when tracking both “success” and “failure”, which is critical for strengthening the opportunities for adaptation learning (Westoby et al. 2020b; McNamara et al. 2022).

Sharing information on poor or limited performance is difficult, especially when funding prospects can be affected, but there are no silver bullets for adaptation, and LLA can challenge business-as-usual and encourage multi-scalar transformations in the culture of the adaptation sector. A key question is how funding structures, power relations and the organisation and implementation of adaptation interventions may open up or close down space for reflective learning processes within organisations as well as deliberative processes within projects. If experimentation, collaboration and deeper learning amongst adaptation actors become a central goal of adaptation projects, rather than delivering measurable material outputs according to usual standards, more equitable and lasting vulnerability reduction may be possible. To support LLA, commitments from existing journals, portals and universities to publish and report findings of poor performance could also reduce stigma, as could “fail forums” that encourage reflective discussions and learning (Westoby et al. 2020b). Researchers could support these efforts by capturing core evidence of ignored lived experiences and benefits of locally led approaches to convince policy- and decision-makers to take the action needed.

## Conclusion

The emergence of LLA, both conceptually and practically, could lead to on-the-ground transformative adaptation where “local” approaches offer benefits that non-local approaches cannot, where local institutions and people control adaptation funds and resources, where access is easier and they have more control over decision-making. Within this process, the key dimensions are inclusion, diverse and democratised knowledges, and equity and justice, all of which also underpin LLA as an idealistic approach (Schipper et al. 2022). LLA draws attention to the need for better standards of participation and representation of diverse local actors and emphasises the challenges of addressing a global problem with local repercussions. Thus, LLA can be seen as a necessary part of addressing unsustainable development—it acknowledges the importance of downward accountability and requires that funders and others who have power report back to communities. Importantly, as a transformative action, LLA is a fundamental ingredient for moving towards greater climate resilient development (Pörtner et al. 2022).

Despite this potential, intersecting Fraser’s three dimensions of injustice with Lukes’ three faces of power to identify the ways in which injustice may persist in LLA, we show that due to competing interests and inequalities in actors’ power, LLA risks reproducing many of the issues that have arisen in earlier adaptation approaches, such as community-based adaptation (see Fig. 1). Distributive injustice persists when finances are still controlled by non-local actors, typically national governments as well as non-government actors, including foreign actors and NGOs/INGOs who refuse to relinquish control over how their funds are allocated and spent. Their control over adaptation finance keeps decision-making power out of the hands of local actors, foreclosing the possibility of moving towards a greater degree of local participation and developing (more) appropriate M&E methods. Recognitional injustice persists when only particular types and levels of “local” actors are involved in decision-making, have a say in how resources are distributed, and in how adaptation success is measured. Representational injustice persists when local participation is mediated by non-local actors, determined by how “local” is defined, resources are distributed, and standards of success are measured.

LLA could retrench existing power dynamics as some actors continue to control the agenda and wield hegemonic power over local actors in decision-making. Furthermore, LLA may fall into the trap of ignoring complexities of the local political economy and of local power structures. If the “local” is perceived as a depoliticised tier, it may reproduce similar challenges and shortcomings of top-down adaptation approaches. Intra-community tensions also raise questions of power and justice that need to be centred in the future LLA initiatives. Marginal groups, including the rural poor, can be trapped within “tightly knitted patron–client networks” (Arens and van Beurden 1977) whereby local elites (with or without support from their external patrons) create a process of “participatory exclusion” or superficial forms of inclusion (Nadiruzzaman and Wrathall 2015). At the same time, individuals and households at the local scale are already creatively adapting to climate change, out of necessity for their lives and livelihoods (Castro and Sen 2022). These actions, along with indigenous-led approaches to addressing climate impacts, could be critical to more effective adaptation, if these actors are leaders in the adaptation process. As maladaptation is a greater possibility when equity and justice issues are not central, LLA needs to be accompanied by a deeper understanding of a community’s underlying vulnerabilities and their drivers in order for it to avoid reproducing or reinforcing existing vulnerabilities or creating new ones.

As large as the promises of LLA are, there are dangers that the concept may facilitate governments to abandon their role in enabling people’s adaptive capacities and instead leave it up to the “local” actors to adapt and fend for themselves. Unless issues of power and justice are at the forefront, LLA risks increasing the onus of adaptation, which already falls on those most affected and those who are least responsible for climate change and have the least resources to address it. Chandler (2020) argues that the community-based or “alternative” approaches to adaptation put forward, which are grounded in local capacity and Indigenous knowledge, put the onus on the “other” (Said, 1987) and do not address power and exploitation. Under the guise of social resilience, the “other” is destined for mere “survival”, without the Western societal transformation around excess. If the funding is largely not reaching those on the ground and will only diminish over time, where does that leave local people who are on the frontlines of climate change and increasingly exposed to climate-induced disasters in a world of excess consumption, capital and mobilities of the West (Chandler et al. 2020)? Ultimately, the utility of the LLA framing in promoting climate justice needs to be tested empirically.

Now is the time to move beyond the rhetoric, incremental shifts and organisational tweaks. We need a new ecosystem that is based on solidarity and is underpinned by shared values and vision (Timson 2020, Hodgson and Isooba 2022). A call for LLA should result in delegating more authority to diverse local actors and covering their core costs, not just passing on the responsibility for achieving results. This should be considered a critical element that contributes to the evaluation of successful LLA initiatives. In doing so, funding, expertise, and resources should be made available to, and be controlled by, local actors as well as disentangled from the values and expectations of funders, with external actors becoming facilitators or enablers. Within this context, there is scope for NGOs, donors, research organisations and consulting companies external to the target communities to (1) offer advice, guidance, and technical support that can then be adapted by local organisations and made contextually relevant, (2) share data with local actors so that they can be empowered to make their own decisions—this could include the outcomes of their LLA initiatives, especially where the initiatives were characterised by poor performance, (3) use their bureaucratic skills to draw attention to the underlying drivers of vulnerability at the local level, and (4) protect local actors from counterproductive incentives and pressures. These actions will help ensure that external actors engage more meaningfully with the dynamics we have outlined in this article and that LLA is done differently and better than business-as-usual adaptation.

## Footnotes

1 The Global Commission on Adaptation was launched in October 2018 with the mandate to “accelerate adaptation by elevating the political visibility of adaptation and focusing on concrete solutions” (https://gca.org/about-us/the-global-commission-on-adaptation/). Following the completion of the mandate of the Commission in 2020, the Global Centre on Adaptation (GCA), through its programmes, has been taking forward the Commission’s action tracks.

2 The eight principles of LLA are as follows: (1) Devolving decision-making to the lowest appropriate level, (2) Addressing structural inequalities faced by vulnerable groups, including women and children, (3) Providing accessible, patient and predictable funding, (4) Investing in local capabilities, (5) Building a robust understanding of climate risk and uncertainty, (6) Flexible programming and learning, (7) Ensuring transparency and accountability, and (8) Collaborative action and investment (Soanes et al. 2021).

3 The Gobeshona Global Conference is an annual conference organised by the International Centre for Climate Change and Development in Bangladesh.
